# Eculizumab for rapidly progressive glomerulonephritis in children

**DOI:** 10.1093/ndt/gfag128

**Published:** 2026-06-16

**Authors:** Luigi Cirillo, Samantha Innocenti, Glenda Cancila, Mariapia Carafa, Valentina Raglianti, Andrea La Tessa, Giulia Sansavini, Carmela Errichiello, Elisa Buti, Augusto Vaglio, Francesca Becherucci, Paola Romagnani

**Affiliations:** Nephrology and Dialysis Unit, Meyer Children’s Hospital IRCCS, Florence, Italy; Department of Biomedical, Experimental and Clinical Sciences “Mario Serio”, University of Florence, Florence, Italy; Nephrology and Dialysis Unit, Meyer Children’s Hospital IRCCS, Florence, Italy; Department of Biomedical, Experimental and Clinical Sciences “Mario Serio”, University of Florence, Florence, Italy; Department of Biomedical, Experimental and Clinical Sciences “Mario Serio”, University of Florence, Florence, Italy; Nephrology and Dialysis Unit, Meyer Children’s Hospital IRCCS, Florence, Italy; Department of Biomedical, Experimental and Clinical Sciences “Mario Serio”, University of Florence, Florence, Italy; Nephrology and Dialysis Unit, Meyer Children’s Hospital IRCCS, Florence, Italy; Nephrology and Dialysis Unit, Meyer Children’s Hospital IRCCS, Florence, Italy; Nephrology and Dialysis Unit, Meyer Children’s Hospital IRCCS, Florence, Italy; Nephrology and Dialysis Unit, Meyer Children’s Hospital IRCCS, Florence, Italy; Nephrology and Dialysis Unit, Meyer Children’s Hospital IRCCS, Florence, Italy; Department of Biomedical, Experimental and Clinical Sciences “Mario Serio”, University of Florence, Florence, Italy; Nephrology and Dialysis Unit, Meyer Children’s Hospital IRCCS, Florence, Italy; Department of Biomedical, Experimental and Clinical Sciences “Mario Serio”, University of Florence, Florence, Italy; Nephrology and Dialysis Unit, Meyer Children’s Hospital IRCCS, Florence, Italy; Department of Biomedical, Experimental and Clinical Sciences “Mario Serio”, University of Florence, Florence, Italy

To the Editor,

Rapidly progressive glomerulonephritis (RPGN) in childhood is rare and time-critical, with high risk of irreversible kidney damage without prompt, targeted intervention [1]. Converging evidence indicates that complement activation can act as a unifying driver of glomerular injury across histologically heterogeneous entities [2, 3]. C5 inhibition with eculizumab offers a biologically plausible strategy to interrupt terminal pathway-mediated injury, yet its use in GN remains off-label and pediatric data are extremely limited. We report a single-center experience of five children with complement-amplified RPGN managed with mechanism-targeted add-on eculizumab in addition to standard care.

We describe five pediatric/adolescent patients (age 5–16 years) treated at the Nephrology and Dialysis Unit of Meyer Children’s Hospital IRCCS (February 2023–June 2025). A rapidly progressive nephritic presentation was operationally defined as (i) rise in serum creatinine ≥2-fold from baseline [estimated glomerular filtration rate (eGFR) reduction >50%] within <4 weeks and (ii) with active urinary sediment [≥20 red blood cells/High-Power Field (RBCs)/HPF and/or ≥10 white blood cells (WBCs)/HPF] and proteinuria. Eculizumab was considered in patients with objective evidence of complement activation—any of the following: low/undetectable serum C3, elevated C3d and/or sC5b-9, C3-dominant immunofluorescence on biopsy, or C3 nephritic factor (C3NeF) positivity. All patients underwent meningococcal prophylaxis per institutional protocol; effective terminal complement blockade was confirmed by CH50 monitoring. Outcomes were prespecified: the primary outcome was return of kidney function to baseline/age-appropriate normal range; secondary outcomes included proteinuria trajectory and resolution of hematuria and sterile leukocyturia. This study was conducted in accordance with the Declaration of Helsinki; formal ethics committee approval was not required per institutional policy for retrospective noninterventional case series.

Five patients (three male and two female; age range 5–16 years) received off-label eculizumab after complete diagnostic work-up including kidney biopsy (Table 1 and Fig. 1). All presented with rapidly deteriorating kidney function (eGFR 11–33 ml/min/1.73 m^2^) and active urinary sediment; 4/5 had nephrotic syndrome at presentation. Complement activation was consistently documented at both systemic and tissue level: C3 was markedly reduced or undetectable in 5/5, with normal C4 in all, and kidney biopsy demonstrated acute proliferative GN with prominent C3 staining on immunofluorescence in 5/5. Additional biomarkers were available in subsets: elevated C3d in 2/5, elevated sC5b-9 in 1/5, and C3NeF positivity in 1/5. Complement genetics identified a chromosome 1 deletion encompassing CFHR3-R1 in one patient.

**Figure 1:**
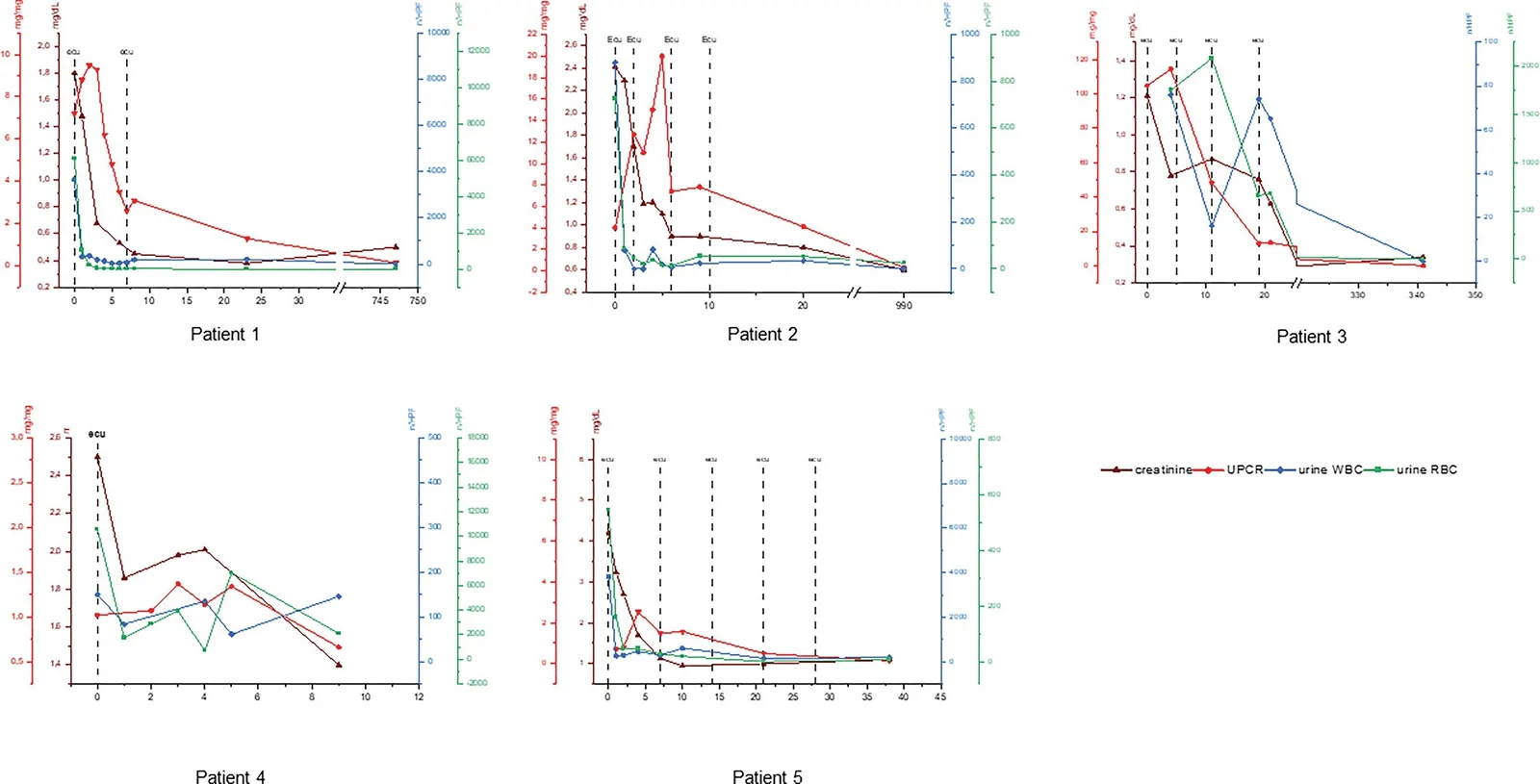
Individual clinical trajectories for each of the five patients following eculizumab initiation (Day 0). For each panel, the left *Y*-axis shows serum creatinine (mg/dl; dark red, triangles) and UPCR (mg/mg; red, circles); the right *Y*-axis shows urine WBC count (*n*/µl; light blue, circles) and urine RBC count (*n*/µl; teal, triangles). Vertical dashed lines indicate eculizumab infusions (labeled “ecu”). Axis breaks (//) indicate time compression between the acute phase and long-term follow-up. Day 0 = date of first eculizumab infusion. UPCR, urine protein-to-creatinine ratio.

**Table 1:** Clinical and laboratory features before and after C5 blockade (eculizumab) and at last follow-up.

Patient	Case 1	Case 2	Case 3	Case 4	Case 5
Pre-eculizumab (baseline; at/just before first dose)					
Sex	M	F	F	M	M
Age (years)	9	5	11	15	16
Diagnosis/biopsy pattern	C3-dominant crescentic GN (C3GN/C3-dominant MPGN phenotype)	Infection-associated proliferative GN during SRNS; C3-dominant IF with IgG co-deposition	Rapidly progressive C3 GN (C3G) with C3NeF positivity	IgAN with C3 co-deposition (proliferative phenotype)	Immune complex-mediated proliferative GN with strong C3 deposition (IgG/IgA/C3)
Concomitant and prior treatments	Supportive care (antibiotics; antihypertensives; loop diuretic); IV methylprednisolone pulses ×3 -> oral prednisone	Steroids at NS onset; rituximab; antimicrobial therapy for pneumonia (steroid escalation limited by T1DM)	IV methylprednisolone pulses ×3; cyclophosphamide; plasmapheresis; antiproteinuric/supportive therapy	IV methylprednisolone pulses ×3 followed by oral steroids	IV methylprednisolone pulses ×3 followed by oral steroids
Time from onset to first eculizumab dose	7 days	13 days	2 months	10 days	2 days
Serum creatinine (mg/dl)	1.88	1.21	2.41	2.50	7.10
eGFR (ml/min/1.73 m^2^)	27	31	22	33	11
C3 (mg/dl)	51	85	Undetectable	130	14
C4 (mg/dl)	33	17	32	14	11
Other complement markers	C3d 76 mU/l; C3NeF negative	sC5b-9 400 ng/ml after four infusions; C3NeF negative	C3d 587 U/l; C3NeF positive	NA	NA
UPCR (mg/mg)	7.2	50	4	1.4	0.7
Urine RBC	4000	7000	726	3900	3825
Urine WBC	7000	105	880	84	545
Eculizumab course (number of infusion)	1^b^	4^a^	4	1^b^	4
Nephrotic syndrome	Yes	Yes	Yes	No	No
Post-eculizumab (early response/end of induction)					
Timing of post-eculizumab assessment	21 days	30 days	30 days	7 days (last follow-up)	30 days
eGFR (ml/min/1.73 m^2^)	93	135	70	47	83
UPCR (mg/mg)	1	4	1.5	0.6	0.5
Urine RBC (n/HPF)	190	16	143	2000	150
Urine WBC (n/HPF)	negative	26	11	100	negative
C3 (mg/dl)	72	159	Undetectable	NA^c^	44
Nephrotic syndrome	resolved	not resolved	resolved	No	No
Last follow-up					
Timing of last follow-up	31 months	12 months	3 years	NA^c^	60 days
eGFR at last follow-up (ml/min/1.73 m^2^)	107	116	100	NA	86
Proteinuria at last follow-up	Absent	Absent (after obinutuzumab)	Absent	NA	Absent
Urine sediment at last follow-up	Absent	Absent	Absent	NA	879 RBC; absent WBC

Urine RBC and WBC are expressed as cells/µl. eGFR is expressed as ml/min/1.73 m^2^.

^a^Eculizumab administered as first disease-targeting agent due to clinical constraints on steroid escalation (concurrent T1DM); corticosteroids initiated the following day.

^b^Single infusion due to rapid clinical and laboratory response.

^c^Follow-up discontinued by family after early improvement; data available at 7 days post-infusion only.

CFHR, complement factor H-related protein; eGFR, estimated glomerular filtration rate (CKiD U25 equation); IC, immune complex; IF, immunofluorescence; MPGN, membranoproliferative glomerulonephritis; NA, not available; sC5b-9, soluble terminal complement complex; SRNS, steroid-resistant nephrotic syndrome; T1DM, type 1 diabetes mellitus; UPCR, urine protein-to-creatinine ratio (mg/mg).

Histologically, 4/5 patients had crescentic involvement; Patient 5 had severe acute kidney injury (AKI) with proliferative immune-complex GN without crescents. Based on immunopathology, cases clustered into two mechanistic patterns: complement-dominant/C3G-spectrum (*n* = 2, including one C3NeF-positive case with relapse) and immune-complex GN with prominent C3 co-activation [*n* = 3: one infection-associated GN, one crescentic IgA nephropathy (IgAN), and one severe AKI with IgG/IgA deposits and strong C3 staining].

Eculizumab was administered as adjunctive therapy in 3/5 patients after prior high-dose corticosteroids (range: 1–4 infusions) and, in selected cases, other immunosuppressants; in Patient 2 eculizumab was the first disease-targeting therapy due to steroid constraints imposed by unmasked type 1 diabetes; in Patient 5 it was administered 1 day before methylprednisolone for logistical reasons, with morning laboratory testing on the day of steroid initiation already showing decline in urinary sediment and serum creatinine. Following initiation of C5 blockade, all patients showed rapid attenuation of urinary inflammatory activity—sterile leukocyturia resolving within days to weeks—and recovery of kidney function. The primary outcome was met in all patients with available longitudinal follow-up: eGFR returned to the normal range (100–135 ml/min/1.73 m^2^ at last assessment). Proteinuria resolved at last follow-up in all patients; urine sediment normalized in all except Case 5, whose last assessment at 60 days showed residual microhematuria. Follow-up ranged from 7 days to 3 years; one patient discontinued care after early improvement. The C3NeF-positive patient (Case 3) relapsed after COVID-19 with recurrence of urinary sediment and subnephrotic proteinuria; eculizumab re-challenge led to renewed resolution of leukocyturia and proteinuria reduction, with normal kidney function at ∼3 years.

Our series illustrates a mechanism-oriented reasoning in which, across histologically heterogeneous entities, objective evidence of complement activation may inform the decision to consider terminal pathway blockade as adjunctive rescue therapy; this framing is hypothesis-generating and does not constitute an evidence-based clinical indication [2, 3]. Within C3G-spectrum disease, the strongest responses occurred in acutely active, crescentic lesions, consistent with prior reports showing more favorable outcomes in highly active terminal-pathway phenotypes compared with chronic, smoldering disease [4, 5]. In infection-related GN, our cases extend sparse pediatric reports of dialysis-threatening presentations reversing after eculizumab, supporting time-limited rescue when complement activation is the dominant acute driver [6, 7]. In the IgA-spectrum case, complement biomarkers helped refine the rescue indication, consistent with evidence that IgAN can engage lectin and alternative pathway activation; anti-C5 outcomes remain heterogeneous in this setting and limited to case reports, supporting a position as short-course rescue only in acutely active, biomarker-positive presentations [8, 9].

Two practical themes emerged. First, timing matters: early deployment during active complement-driven injury may favor reversibility before structural damage is established, a pattern reflected in the complete renal recovery observed across our cohort. Second, a multiparametric biomarker panel (tissue + urine + serum) may support case-by-case reasoning [10]; within this framework, re-challenge can be considered in biomarker-positive relapses driven by intercurrent infections or persistent upstream drivers (e.g. C3NeF positivity and CFHR1/R3 rearrangement), provided baseline measures and risk mitigation are revisited. Limitations include the uncontrolled design, small sample size, histologic heterogeneity, selection bias, incomplete complement profiling (markers and genetics not uniformly obtained), and heterogeneous previous immunosuppression limiting causal attribution; no causal inference regarding C5 blockade can therefore be drawn from this series. Notably, all patients with available longitudinal follow-up achieved a favorable renal outcome, which contrasts with the unsatisfactory prognosis reported in published pediatric RPGN series [1]. Meningococcal prophylaxis and infection exclusion prior to treatment are mandatory safety obligations that apply regardless of the product used. On the other side of this equation, the availability of eculizumab biosimilars may substantially improve affordability and broaden access to complement-targeted rescue in resource-constrained settings, potentially enabling timely treatment otherwise precluded by cost. Prospective, biomarker-enriched pediatric studies are needed to establish optimal timing, duration, and re-challenge criteria for complement-targeted rescue in pediatric RPGN.

## Supplementary Material

gfag128_Supplemental_File

## References

[1] Meena J, Nangla P, Ojha S et al. Etiology, clinical profile, and outcomes of crescentic glomerulonephritis in children: a systematic review. Pediatr Nephrol. 2025;40:675–84. 10.1007/s00467-024-06521-3 39278987

[2] Anders H-J, Kitching AR, Leung N et al. Glomerulonephritis: immunopathogenesis and immunotherapy. Nat Rev Immunol. 2023;23:453–71. 10.1038/s41577-022-00816-y PMC9838307 36635359

[3] Petr V, Thurman JM. The role of complement in kidney disease. Nat Rev Nephrol. 2023;19:771–87. 10.1038/s41581-023-00766-1 37735215

[4] Le Quintrec M, Lapeyraque A-L, Lionet A et al. Patterns of clinical response to eculizumab in patients with C3 glomerulopathy. Am J Kidney Dis. 2018;72:84–92. 10.1053/j.ajkd.2017.11.019 29429752

[5] Le Quintrec M, Lionet A, Kandel C et al. Eculizumab for treatment of rapidly progressive C3 glomerulopathy. Am J Kidney Dis. 2015;65:484–9. 10.1053/j.ajkd.2014.09.025 25530108

[6] Chehade H, Rotman S, Frémeaux-Bacchi V et al. Blockade of C5 in severe acute postinfectious glomerulonephritis associated with anti-factor H autoantibody. Am J Kidney Dis. 2016;68:944–8. 10.1053/j.ajkd.2016.06.026 27683044

[7] Chehade H, Guzzo G, Cachat F et al. Eculizumab as a new treatment for severe acute post-infectious glomerulonephritis: two case reports. Front Med. 2021;8:663258. 10.3389/fmed.2021.663258 PMC8350112 34381795

[8] Caravaca-Fontán F, Gutiérrez E, Sevillano ÁM et al. Targeting complement in IgA nephropathy. Clin Kidney J. 2023;16:ii28–39. 10.1093/ckj/sfad198 PMC10695513 38053977

[9] Barratt J, Lafayette RA, Zhang H et al. IgA nephropathy: the lectin pathway and implications for targeted therapy. Kidney Int. 2023;104:254–64. 10.1016/j.kint.2023.04.029 37263354

[10] Rizk DV, Dixon BP, Chan M et al. Biomarkers in the management of complement-mediated kidney diseases in the era of complement therapeutics. Clin J Am Soc Nephrol. 2025. 10.2215/CJN.0000000967 PMC13379139 41404698

