# Supplementary material for: Eculizumab for rapidly progressive glomerulonephritis in children
Source: Nephrol Dial Transplant. 2026 Jun 16;41(10):1919–22. doi: 10.1093/ndt/gfag128 (PMC13624806; doi:10.1093/ndt/gfag128)
Supplement: Supplementary file 1 [file gfag128_supplemental_file.docx]

**Eculizumab for rapidly progressive glomerulonephritis in children**

**Individual Case Descriptions**

**Case 1**

A 9-year-old male presented to the ED with periorbital edema, fever, and gross hematuria; one month earlier, he had had a cough and rhinitis treated with corticosteroids and salbutamol. On admission, he showed hypertension and weight gain despite apparently preserved urine output. Labs revealed AKI (serum creatinine 1.52 mg/dL; urea 83 mg/dL), hypoproteinemia (total protein 5.34 g/dL; albumin 28 g/L), mild leukocytosis with normal differential, negative CRP and procalcitonin. Given the recent upper respiratory tract infection, ASO titer and complementemia were evaluated. ASO was mildly positive (ASO titer 880 IU/mL) with low C3 (below local reference), normal C4 and increased C3d 76 mU/L (normal <55). Urinalysis documented gross hematuria and nephrotic-range proteinuria (~400 mg/dL on dipstick) with an extremely active sediment before complement blockade (~7,000 leukocytes/μL and ~4,000 erythrocytes/μL). Empiric amoxicillin–clavulanate, amlodipine, and furosemide were started; over the first 72 h, renal function worsened and proteinuria rose to 5 g/day, with new lower-limb edema. Because of rapid progression, kidney biopsy was performed and showed mesangial and endocapillary proliferation with crescent formation, together with a mixed acute and chronic inflammatory infiltrate involving tubules, interstitium, and glomeruli; immunopathology demonstrated dominant C3 deposition (C3-predominant pattern), consistent with complement-driven RPGN; crescents were present in 55% of glomeruli. After one week of hospitalisation and supportive care, suboptimal blood pressure control prompted the addition of doxazosin, and worsening of proteinuria occurred. Infectious screening was negative; autoimmunity was positive only for ANA 1:640 (nucleolar pattern) and anti-fibrillarin antibodies, which were not confirmed on subsequent testing and lacked clinical correlates of systemic sclerosis over 31 months of follow-up. The patient then received three intravenous methylprednisolone pulses followed by oral prednisone; given persistent renal dysfunction with ongoing hypocomplementemia and biopsy evidence of complement activation, off-label eculizumab was initiated after exclusion of active infection. Within 24 hours urine output and kidney function improved markedly and diuretics were no longer required; renal function normalized in just under one week, the urinary inflammatory activity rapidly abated with leukocytes falling to zero by one week, and hematuria decreased in parallel, followed by a sustained decline in proteinuria. At discharge eculizumab was withdrawn after 2 doses, and a steroid taper was prescribed; mycophenolate mofetil, briefly overlapped, was discontinued once C3 nephritic factor testing returned negative. Complement genetic testing revealed no pathogenic variants but identified polymorphisms previously associated with complement dysregulation. After 31 months of follow-up kidney function is normal.

**Case 2**

A 5-year-old girl with steroid-resistant nephrotic syndrome^1^—in whom type 1 diabetes mellitus was unmasked during initial corticosteroid therapy—had a biopsy and serology demonstrating anti-nephrin and anti-podocin antibodies, for which she received rituximab with a marked but incomplete reduction in proteinuria. Her course was complicated by Chlamydia pneumoniae pneumonia; during this infectious episode, she developed an abrupt rise in serum creatinine with reduced urine output, diuretic-resistant edema, and hypertension. Urinalysis showed worsening proteinuria with new onset of microhematuria and leukocyturia. Complement workup revealed low C3 and a markedly elevated soluble C5b-9 (400 ng/mL; normal values <239 ng/mL) as a sign of complement activation. Because of rapidly progressive glomerulonephritis, a kidney biopsy was performed and showed mesangial and endocapillary proliferation with a cellular crescent in one glomerulus (~10% of glomeruli); immunofluorescence demonstrated dominant C3 deposition with IgG co-deposition. Suspecting acute infection-related GN driven by complement activation—and considering the constraint on steroid escalation imposed by type 1 diabetes—off-label eculizumab was administered as rescue therapy (after initiation of treatment for the concurrent infectious disease and meningococcal prophylaxis, discussion and informed consent). Following infusion, renal function improved promptly, microhematuria and leukocyturia decreased, urine output increased, and edema regressed. Renal function subsequently normalized over the ensuing month, so eculizumab was discontinued after 4 doses. Because anti-nephrin and anti-podocin antibodies remained detectable in patient serum in association with persistent proteinuria, rituximab response was considered incomplete, and the patient was later treated with obinutuzumab, achieving complete remission of nephrotic syndrome. After 12 months of follow-up kidney function is normal.

**Case 3**

A previously healthy 11-year-old female was referred to the emergency department (ED) for periorbital, pretibial edema and hypertension. Routine laboratory testing was consistent with nephrotic syndrome and increased serum creatinine with C3 below the assay limit; urinalysis showed nephrotic-range proteinuria, microhematuria, and sterile leukocyturia. At 48 h follow-up, kidney function rapidly decreased up to values of serum creatinine over 2 mg/dl, and fluid overload was partially responsive to furosemide. Renal biopsy showed diffuse mesangial and endocapillary proliferation with crescents and a chronic periglomerular inflammatory infiltrate; crescents were present in 35% of glomeruli; immunofluorescence demonstrated massive C3 deposition. Histology and laboratory findings were suggestive of a rapidly progressive C3 glomerulonephritis, so the patient received three methylprednisolone pulses and cyclophosphamide for worsening renal injury, with limited effect. Complement studies confirmed complement activation with increased C3d (587 U/L) and C3 nephritic factor (C3NeF) positivity. Given C3NeF positivity, plasmapheresis was initiated; however, C3 remained undetectable and renal function deteriorated further, with serum creatinine doubling two months after onset. After discussion and consent, off-label eculizumab was started, with substantial clinical improvement and near-normalisation of creatinine within two weeks. Eculizumab was tapered and discontinued over four months; at withdrawal, urinalysis was negative while C3 remained undetectable. Antiproteinuric therapy was continued, and mycophenolate mofetil was introduced in view of C3NeF positivity. Genetic testing identified a chromosome 1 deletion encompassing complement-related genes (CFHR3-R1). Three months after discontinuation, the patient developed COVID-19 with recurrence of microhematuria, sterile leukocyturia, and sub-nephrotic proteinuria up to 1.8 g/day; eculizumab was re-initiated, leading again to resolution of leukocyturia and microhematuria and reduction of proteinuria to ~500 mg/day. The drug was subsequently stopped, antiproteinuric therapy was maintained, and mycophenolate was later withdrawn without clinical or laboratory worsening. After three years of follow-up the renal function is normal without proteinuria and no sediment.

**Case 4**

A previously healthy 15-year-old boy presented to the emergency department for gross hematuria. Urinalysis confirmed microhematuria, sub-nephrotic proteinuria, and sterile leukocyturia; blood tests showed acute kidney injury. Given diagnostic uncertainty and rapid deterioration of renal function, a kidney biopsy was performed. Immunofluorescence demonstrated mesangial and capillary-wall deposits of IgA and C3 with lesser IgG co-deposition, consistent with IgA nephropathy; light microscopy revealed mesangial and endocapillary hypercellularity with a lobulated (flocculonodular) pattern and interstitial inflammatory infiltrates, with extracapillary crescent formation in ~10% of glomeruli. After standard serologic and microbiologic evaluations, the patient received three days of intravenous methylprednisolone pulses, followed by oral steroids. Because renal function failed to improve over ten days, off-label eculizumab was administered after informed consent; the decision was based on the biopsy evidence of C3 co-deposition alongside crescentic involvement, the lack of response to corticosteroids, and the need for a rapidly acting intervention to interrupt ongoing glomerular injury, given the progressive deterioration of renal function. Systemic complement biomarkers (C3d, sC5b-9) were not available at the time of the clinical decision. The infusion was well tolerated. In the ensuing days, leukocyturia resolved, with concomitant improvement in proteinuria and kidney function. The family subsequently discontinued follow-up at our centre after early improvement.

**Case 5**

A 16-year-old boy presented to the ED after 3 days of frequent non-bilious vomiting (≈7 episodes/day) associated with poor oral intake and crampy epigastric pain; during the first 48 hours he had fever up to 38.9 °C treated with paracetamol and took a single dose of ibuprofen for headache. He reported reduced urine output. On examination he was in good general condition and hemodynamically stable, afebrile, with clinical signs of dehydration. Initial blood tests showed severe acute kidney injury (serum creatinine (7.1 mg/dL); urea (180 mg/dL) and inflammatory activation (WBC 12,000/mm³ with neutrophilia; CRP 7 mg/L), with mild hyponatremia and mild transaminase elevation. Urinalysis revealed proteinuria (dipstick 3+; UPCR 0.7 mg/mg), hematuria (dipstick blood 3+; RBC 3825/µL), and sterile leukocyturia (WBC 545/µL). Abdominal ultrasound documented normal-sized kidneys with preserved corticomedullary differentiation and no urinary tract obstruction. Despite intravenous rehydration, renal function remained markedly impaired on repeat testing, and oliguria persisted, prompting admission for management of severe AKI with active urinary sediment.

On hospital day 1, complement testing showed marked hypocomplementemia with very low C3 (14 mg/dL); and normal C4, supporting a working diagnosis of hypocomplementemic immune-mediated glomerulonephritis; additional baseline studies were obtained, including infectious screening and autoimmune panel. Given the severity of renal dysfunction and the active urinary sediment, a kidney biopsy was performed, demonstrating an acute proliferative glomerulonephritis with mesangial, endocapillary proliferation without crescents; immunofluorescence showed IgG (++), IgA (++), and strong C3 (+++) deposition with kappa/lambda and C1q (+/−), consistent with an immune complex–mediated proliferative GN with prominent complement activation.

Supportive care included carefully titrated intravenous fluids with oral fluid restriction and loop diuretics adjusted according to urine output. In view of the biopsy findings together with ongoing complement consumption, off-label eculizumab was initiated after informed consent and meningococcal risk management per institutional protocol (antibiotic prophylaxis initiated given incomplete vaccination schedule). High-dose steroids were started the following day for logistical reasons related to pharmacy preparation, then transitioned to oral prednisone 60 mg/day. Over the ensuing days, renal function began to improve accompanied by increasing urine output and reduction of edema; however, C3 remained reduced (44 mg/dL). The patient completed a full induction course of eculizumab. At approximately 30 days from symptom onset, renal function had returned to normal, while C3 remained reduced. Urinalysis showed residual sub-nephrotic proteinuria (UPCR ~1 mg/mg) with only scant hematuria, and resolution of leukocyturia. After approximately 60 days of follow-up, eGFR had recovered to 80 mL/min/1.73m², with residual sub-nephrotic proteinuria (UPCR approximately 1 mg/mg) and microhematuria.

Supplementary Bibliography

1. Raglianti V, Cirillo L, Angelotti ML, De Chiara L, Mazzinghi B, Antonelli G, Conte C, Melica ME, Peired AJ, Lazzeri E, Lasagni L, Palazzo V, Landini S, Buccoliero AM, Innocenti S, Errichiello C, Buti E, Sansavini G, La Tessa A, Becherucci F, Anders HJ, Romagnani P. Anti-podocin Enzyme-Linked Immunosorbent Assay Guides Immunotherapy in Steroid-Resistant Nephrotic Syndrome. Kidney Int Rep. 2025 Jul 16;10(10):3689-3693.
